# Insights into the heterogeneity of iNKT cells: tissue-resident and circulating subsets shaped by local microenvironmental cues

**DOI:** 10.3389/fimmu.2024.1349184

**Published:** 2024-02-19

**Authors:** Guangwei Cui, Shinya Abe, Ryoma Kato, Koichi Ikuta

**Affiliations:** ^1^Laboratory of Immune Regulation, Department of Virus Research, Institute for Life and Medical Sciences, Kyoto University, Kyoto, Japan; ^2^Faculty of Pharmaceutical Sciences, Kyoto University, Kyoto, Japan

**Keywords:** iNKT cell, heterogeneity, immune microenvironment, IL-15, immunotherapy

## Abstract

Invariant natural killer T (iNKT) cells are a distinct subpopulation of innate-like T lymphocytes. They are characterized by semi-invariant T cell receptors (TCRs) that recognize both self and foreign lipid antigens presented by CD1d, a non-polymorphic MHC class I-like molecule. iNKT cells play a critical role in stimulating innate and adaptive immune responses, providing an effective defense against infections and cancers, while also contributing to chronic inflammation. The functions of iNKT cells are specific to their location, ranging from lymphoid to non-lymphoid tissues, such as the thymus, lung, liver, intestine, and adipose tissue. This review aims to provide insights into the heterogeneity of development and function in iNKT cells. First, we will review the expression of master transcription factors that define subsets of iNKT cells and their production of effector molecules such as cytokines and granzymes. In this article, we describe the gene expression profiles contributing to the kinetics, distribution, and cytotoxicity of iNKT cells across different tissue types. We also review the impact of cytokine production in distinct immune microenvironments on iNKT cell heterogeneity, highlighting a recently identified circulating iNKT cell subset. Additionally, we explore the potential of exploiting iNKT cell heterogeneity to create potent immunotherapies for human cancers in the future.

## Introduction

Invariant natural killer T (iNKT) cells are a subset of T lymphocytes with innate-like properties that detect self or foreign lipid antigens presented on non-polymorphic major histocompatibility complex (MHC) class I-like molecule CD1d (1–3). They express semi-invariant T cell receptors (TCRs) encoded by Vα14-Jα18 in mice and Vα24-Jα18 in humans, paired with a limited number of TCRβ chains Vβ8.2, Vβ7, or Vβ2 in mice and Vβ11 in humans (4). iNKT cells become activated rapidly at the onset of immune responses by signals from CD1d-restricted semi-invariant TCRs and cytokine receptors such as the interleukin-12 receptor (IL-12R) and IL-18R (5). Activated iNKT cells exert their effector functions indirectly by inducing immune responses in other immune cells and directly through cytotoxicity against target cells.

Upon activation, iNKT cells rapidly secrete significant levels of cytokines, such as interferon-γ (IFN-γ) and IL-4. These cytokines promote the maturation of antigen-presenting cells (APCs), NK cells, and cytotoxic T lymphocytes (CTLs) (6, 7). Additional cytokines such as IL-2, IL-5, IL-6, IL-10, IL-13, IL-17, and IL-22, along with growth factors and chemokines (CCL3, CCL4, and CCL5), are produced by iNKT cells to enhance immune responses (8). Additionally, iNKT cells can stimulate the maturation and activation of APCs through their interaction between CD40 and CD40L. Regarding direct cytotoxicity, activated iNKT cells release cytotoxic proteins, such as granzymes and perforin, which collaborate to provoke apoptosis of target cells. Furthermore, iNKT cells express Fas ligand (FASL) and tumor necrosis factor (TNF)-related apoptosis-inducing ligand (TRAIL), which can trigger target cell apoptosis by activating the death receptor pathway (3, 9).

Due to their numerous effector functions, iNKT cells link innate and adaptive immunity, rendering them vital for host defense against cancer and infection. This versatility makes them an attractive target for immunotherapy (10). However, as most iNKT cells are tissue-resident, their regulation and effector functions vary across tissues. Several studies have demonstrated that iNKT cells in mice exhibit heterogeneity and perform diverse functions and that the composition of iNKT cell subsets can alter disease outcomes (11–14). This article reviews the tissue residency and heterogeneity of iNKT cells across different tissues, focusing on a newly identified circulating iNKT cell subset that differs from conventional tissue-resident iNKT cells.

## Developmental and functional subsets of iNKT cells

Like conventional T cells, most iNKT cells arise from CD4^+^CD8^+^ double-positive (DP) thymocytes in the thymus and are regulated by CD1d-expressing DP thymocytes, as well as the crucial cytokine IL-7 (1, 15–18). Alternatively, it has been reported that a fraction of CD4^−^CD8^−^ double-negative (DN) iNKT cells develop from late DN-stage thymocytes (19). The development of iNKT cells is regulated by the transcription factors PLZF (encoded by *Zbtb16*) and EGR2 (20, 21). Specifically, the absence of PLZF results in a significant decrease in the number of iNKT cells, and bone marrow chimeric mice deficient in EGR2 show an arrest of iNKT cell differentiation.

Mouse iNKT cells have been categorized into four developmental subsets, namely stage 0, 1, 2, and 3 iNKT cells, and three effector subsets, namely NKT1, NKT2, and NKT17 cells (2, 7, 22–25). During thymic development in mice, iNKT cells can be categorized into four subsets using CD24, CD44, and NK1.1 markers. iNKT cells undergo positive selection during the earliest stage 0 precursor cells (CD24^high^CD44^−^NK1.1^−^) in response to agonistic interactions with CD4^+^CD8^+^ double-positive (DP) thymocytes in the thymic cortex. Then, CD24 is downregulated in iNKT cells to transit into the immature stage 1 (CD24^low^CD44^−^NK1.1^−^) cells. Subsequently, the cells progress to stage 2 (CD24^low^CD44^+^NK1.1^−^) cells with upregulation of CD44 and eventually differentiate into mature stage 3 (CD24^low^CD44^+^NK1.1^+^) cells. The mature population of iNKT cells displays functional heterogeneity. These cells have been categorized into three effector subsets, NKT1, NKT2, and NKT17 cells, based on differential expression of cytokines and transcription factors associated with distinct functions similar to Th1, Th2, and Th17 cells in helper T cells, respectively (7, 24–27). Generally, NKT1 cells are T-bet^+^PLZF^low^ and produce IFN-γ, NKT2 cells are GATA-3^+^PLZF^high^ and produce IL-4, and NKT17 cells are RORγt^+^PLZF^int^ and produce IL-17. Notably, NKT1, NKT2, and NKT17 cells are found in different fractions of stage 3, stage 1 or 2, and stage 2, respectively, suggesting that markers of conventional iNKT cell developmental stages in the thymus, CD44 and NK1.1, are expressed heterogeneously across these distinct effector subsets (7). Functional subsets of iNKT cells in C57BL/6 mice can be distinguished by NK1.1, CD4, and IL-17RB cell surface markers. Specifically, NK1.1^+^ iNKT cells typically produce IFN-γ and are categorized as NKT1 cells. NKT2 and NKT17 cells, distinguished by CD4^+^ and CD4^−^ expression, respectively, express IL-17RB, while NKT1 cells do not express this cytokine receptor. CD122 (IL-2Rβ) and CXCR3 are useful surface markers for NKT1 cells (7, 25), as other mouse strains, except for C57BL/6, do not express NK1.1. Furthermore, CD138 is uniquely expressed in NKT17 cells among effector subsets of iNKT cells (28–30). In addition to NKT1, NKT2, and NKT17, a population of iNKT cells has been identified as follicular helper NKT (NKT_FH_) cells that produce IL-21 and provide cognate help to antigen-specific B cells (31). These iNKT cells express BCL-6, CXCR5, and PD-1, similar to follicular helper T cells (T_FH_). Moreover, IL-10-producing NKT10 cells express E4BP4 but not FoxP3 (32). Upon stimulation, they exhibit regulatory properties akin to Treg cells.

Certain iNKT cells acquire various NK cell receptors and generate cytotoxic molecules similar to NK cells. The development of iNKT cells, especially NKT1 cells, is regulated by IL-15, an essential cytokine for NK cells (11, 33). Since medullary thymic epithelial cells (mTECs) are the primary source of IL-15 and the diversity of mTECs controls intrathymic iNKT cell development and maturation, it has been proposed that the thymic medulla is crucial for the continued development and maturation of iNKT cells (34–36). Subsequently, it was discovered that CCR7^+^PLZF^high^ iNKT cells in the mouse thymus serve as progenitors for the three effector subsets of NKT1, NKT2, and NKT17 cells in the mouse. Furthermore, CCR7 enables the progenitors to migrate from the cortex to the medulla, and iNKT cells expressing CCR7 leave the thymus in a Klf2- and S1PR1-dependent manner, followed by subsequent maturation and expansion in peripheral tissues (12, 37).

Human iNKT cells are typically divided into two subsets: CD4^+^ and CD4^−^ iNKT cells (38, 39). CD4^−^ iNKT cells include CD4^−^CD8^−^ DN and CD8^+^ iNKT cells. CD4^+^ iNKT cells produce Th1 and Th2 cytokines, such as IFN-γ, IL-4 and IL-13, while CD4^−^ iNKT cells mainly produce Th1 cytokines, such as IFN-γ and TNF-α. Upon activation, human CD4^−^ iNKT cells, particularly DN iNKT cells, exhibit greater effector functions and cytolytic activity, with increased IFN-γ expression similar to NK cells. Additionally, in the presence of TGF-β and rapamycin, some human iNKT cells express Foxp3 and exhibit Treg-like features. They are functionally capable of suppressing the proliferation of conventional CD4^+^ T cells by producing IL-10 (40).

## Tissue-residency and heterogeneity of iNKT cells

Based on the results of thymus transplantation and parabiosis experiments in mice, iNKT cells are considered tissue-resident lymphocytes (11, 41–43). These cells reside permanently within various lymphoid and non-lymphoid organs, such as the thymus, spleen, lymph nodes, liver, lung, intestine, and adipose tissue. iNKT cells play a crucial role in both systemic and local immunity. RNA-seq analysis revealed the differences in tissue location between effector subsets of iNKT cells (44). A recent study utilizing single-cell RNA sequencing (scRNA-seq) investigated iNKT cells in different organs (12). The study integrated thymic iNKT cells with peripheral iNKT cells and distinguished iNKT cells at various developmental stages in the thymus and across different peripheral tissues using the uniform manifold approximation and projection (UMAP) plot. The iNKT cells isolated from different organs exhibit unique tissue-specific features dependent on their origin. Furthermore, while iNKT cells are scarce in each organ under normal conditions, their numbers significantly increase during inflammation and infection (45, 46). Therefore, iNKT cells are molded by diverse tissue microenvironments and display phenotypic variety influenced by their resident tissues and effector subtypes (**Table 1**).

**Table 1:** Heterogeneity of tissue-resident iNKT cells in mice.

Tissue	Localization	Feature
**Thymus**	Matured cells distribute in the thymic medulla	• NKT1 and NKT2: phenotypic and functional heterogeneity (47) • NKT17: relatively uniform (47) • Require CD28 co-stimulation (48)
**Liver**	Patrol along the liver sinusoids	• Activated by CD1d-dependent lipid antigen presentation (49) • CD1d and IL-7 dependent cell maintenance (49, 50) • Higher expression of Bcl-2 and Socs2 (12) • Higher proliferation status (12) • Related to hepatocyte cell death and liver damage (14, 49) • LFA-1, CXCR6, and CD69 for residence and maintenance (41, 51–54) • NKT17: promote cancer cell extravasation (55)
**Lung**	Vasculature and interstitial tissue	• Exacerbate inflammation in allergies (45, 56) • Th2 cytokine expression (45, 56) • ICOS-ICOSL dependent cytokine production (56) • Enriched transcripts of bZIP and NF-kB family (44) • CCR4, CCR9, and CXCR6 for migration and maintenance (41, 57)
**Gut**	Prevalent in the lamina propria than in the epithelial layer	• Activated by dietary components, with microbial metabolites containing lipid molecules (58) • Modulate gut inflammation, tissue homeostasis, and microbiota (59–61) • Impacted by commensal microbiota (62–64)
**Adipose tissue**	-	• Frequency of NKT10 cells is significantly high (32) • Regulate the anti-inflammatory immune cells, such as M2 macrophages and Treg cells (65, 66) • Higher expression of GLUT1 and CD36 for glycolytic and fatty acid metabolism (67) • Metabolically active and require AMPK pathway to regulate adipose tissue homeostasis during inflammation (67)

Although effector subsets of iNKT cells, namely NKT1, NKT2, and NKT17, differentiated in the thymus, most mature iNKT cells found in the mouse thymus are thymus-resident (11, 43, 68, 69). A recent scRNA-seq study revealed that NKT1 and NKT2 cells exhibit considerable phenotypic and functional heterogeneity, while NKT17 cells are relatively uniform (47). Apart from differences among effector subsets of iNKT cells, thymus-resident iNKT cells require CD28 co-stimulation during antigenic activation (48). In addition, local signals seem to influence the TCR repertoire and antigen specificity of tissue-resident iNKT cells. These iNKT cells exhibit diverse TCR Vβ usage and experience clonal expansion in the thymus, spleen, and lymph nodes. Furthermore, distinct TCRβ repertoires in iNKT cells from different tissues are associated with their different ability to recognize lipid antigens (70).

In mice, iNKT cells are most abundant in the liver compared to other tissues, while in humans, they are also enriched but much less abundant. Hepatic iNKT cells conduct random and crawling patrols along the liver sinusoids. In mice, the patrolling iNKT cells in the liver can be arrested through TCR signals, inflammatory cytokines like IL-12 and IL-18, or microbial antigens during infections (71, 72). Sinusoidal endothelial cells, Kupffer cells, stellate cells, and hepatocytes all express CD1d, presenting lipid antigens to hepatic iNKT cells, and CD1d expression on hepatocytes contributes to maintaining hepatic iNKT cells (49). Besides CD1d, hepatocyte-derived IL-7 is crucial for iNKT cell survival in the liver but does not affect iNKT cells in the spleen (50). The anti-apoptotic gene *Bcl2* and suppressor of cytokine signaling 2 (Socs2) are highly expressed in hepatic iNKT cells, when compared to iNKT cells in the spleen and lymph nodes. In addition, hepatic NKT1 cells show a higher cell proliferation status (12). By producing IFN-γ, hepatic iNKT cells defend against infections of *B. burgdorferi*, hepatitis B virus (HBV), and hepatitis C virus (HCV) (73–76).

Activated hepatic iNKT cells have been implicated in liver damage and fibrosis in pathological conditions like hepatitis, chronic inflammation, and sterile liver injury, in addition to their immunomodulatory roles. For instance, hepatic iNKT cells can induce FASL expression and release TNF, perforin, and granzymes, leading to hepatocyte cell death (14, 49). Liver-resident NKT17 cells secrete IL-22, which has been found to promote cancer cell extravasation during liver metastasis (55). Additionally, iNKT cells found in the liver, spleen, and mucosal tissues express P2RX7, an ATP-sensitive purinergic receptor that plays a crucial role in the survival of tissue-resident lymphocytes (77, 78). In the liver, the depletion of iNKT cells, dependent on P2RX7, functions as a feedback mechanism to limit tissue damage related to iNKT cells, leading to a tolerogenic microenvironment.

iNKT cells are present in the lung’s vasculature and interstitial tissues in mice, contributing to an effective immune response against pulmonary infections (79–81). They are essential in protecting against viral and bacterial infections, often activated in a CD1d-dependent manner, with cytokine production, such as IFN-γ or IL-17. Conversely, activated pulmonary iNKT cells have also been shown to exacerbate inflammation in allergies. Studies utilizing the OVA-induced model of allergic asthma have reported that iNKT cells produce IL-4, IL-5, IL-10, IL-13, and IFN-γ in an inducible T cell costimulator (ICOS)-ICOS ligand (ICOSL)-dependent manner (45, 56). Transcripts encoding AP-1 and other members of the basic leucine zipper (bZIP) family, as well as some members of the NF-κB family, are enriched in lung iNKT cell subsets, as are transcripts encoding CTLA-4, CD69, and Nur77 (44).

In mouse and human gut, iNKT cells are more prevalent in the lamina propria than in the epithelial layer (62, 82). These cells can be activated and impacted by dietary components, with microbial metabolites containing lipid molecules presented by CD1d (58). Intestinal iNKT cells emerge prior to microbial colonization and contribute to enhancing or suppressing immune responses. They modulate gut inflammation, tissue homeostasis, and microbiota by binding with CD1d and expressing IFN-γ (59–61). On the other hand, the commensal microbiota impacts iNKT cells (62, 63). For example, intestinal iNKT cell numbers are brought back to normal levels in neonatal germ-free mice through monocolonization with *B. fragilis* or exposure to the purified glycolipid antigen of this bacterium (64). Furthermore, environmental differences can modulate the frequency, Vβ usage, and cytokine production of intestinal iNKT cells in mice, despite expressing CD69, CD44, and CD122, similarly to their splenic counterparts (62).

iNKT cells are enriched in the visceral adipose tissue (VAT) in mice and humans (32, 65, 66). The frequency of IL-10-producing NKT10 cells is significantly high in the iNKT cells in the adipose tissue. Therefore, the tissue-resident iNKT cells in the adipose tissue regulate the homeostasis of anti-inflammatory immune cells, such as M2 macrophages and Treg cells, and maintain tissue quiescence. Furthermore, stimulating iNKT cells in the adipose tissue *in vivo* leads to the expansion of iNKT cells and the production of IL-10 and IL-4, which can induce M2 macrophages (65). iNKT cells interact with M2 macrophages, resulting in IL-4 and IL-13 production, while interaction with M1 macrophages triggers IFN-γ production by iNKT cells. A recent study showed that proteins necessary for glycolytic and fatty acid metabolism, such as GLUT1 and CD36, are higher in the adipose tissue iNKT cells than in hepatic and spleen iNKT cells (67). Additionally, adipose tissue iNKT cells are metabolically active and require the AMP-activated protein kinase (AMPK) pathway to regulate adipose tissue homeostasis during inflammation induced by obesity.

In addition, chemoattractants, integrins, and lectins drive tissue-specific differences in mouse iNKT cells. The CXC chemokine receptor 5 (CXCR5) is expressed by a subset of iNKT cells in the spleen, leading to homing to lymphoid organs in response to CXCL13 (83). For iNKT cells in the lung, their migration or maintenance heavily depends on several chemokine receptors, including CCR4, CCR9, and CXCR6 (41, 57). PLZF, a critical transcription factor for iNKT cell development, upregulates LFA-1 in the liver for the intravascular residence of hepatic iNKT cells (41). Furthermore, CXCR6 and Id2 play a critical role in the accumulation and maintenance of hepatic iNKT cells (51, 52). CD69, a C-type lectin receptor, which inhibits S1PR1 and is vital for tissue residency of T cells, is increased on non-circulating iNKT cells within the liver (53, 54). More recently, a study examined the establishment of tissue residency following lineage commitment of iNKT cells. The study classified iNKT cells into three populations: resident memory (iNKT_RM_, CD69^+^CD62L^−^), effector memory (iNKT_EM_, CD69^−^CD62L^−^), and central memory (iNKT_CM_, CD69^−^CD62L^+^) cells using the C-type lectin receptor CD69 and the lymph node homing receptor CD62L (84). Hobit and its homolog Blimp-1 function as major regulators of tissue retention across various tissue-resident lymphocytes. Hobit expression is increased in iNKT_RM_ cells with high expression of tissue-resident molecules, such as CD49a, CXCR6, and P2RX7. This implies that Hobit and Blimp-1 direct not only iNKT cell differentiation but also tissue-resident features.

Furthermore, studies have examined the significance of metabolism in T cell development, proliferation, and effector functions (85). Concerning iNKT cells, maintaining elevated levels of oxidative phosphorylation (OXPHOS) seems to be crucial for sustaining iNKT cell survival, while glutaminolysis may contribute to the regulation of activation-induced iNKT cell expansion in mice (86). Additionally, the regulation of tissue-specific immunometabolism in mouse iNKT cells has been found to impact liver injury and inflammation caused by obesity (67). Nevertheless, the effects of nutrient availability in the tissue microenvironment on the metabolic programming of tissue-resident iNKT cells at steady state remain largely unknown. Although iNKT cells are infrequent *in vivo*, a new flow cytometry-based single-cell metabolism profiling technique used to study γδ T cell and ILC2 metabolism may be able to analyze the metabolic needs of diverse tissue-resident iNKT cells in the future (87, 88). Consequently, various local tissue factors related to cell distribution, differentiation, survival, activation, and metabolism contribute to the heterogeneity of iNKT cells.

## Thymic IL-15 niche-dependent circulating iNKT cells

Coordinated cytokine expression by diverse stromal cells in tissue-specific microenvironments regulates the distinct lymphocyte development and immune response in various tissues. Specifically, IL-15 is a crucial cytokine for the development, maintenance, and function of iNKT cells in the thymus and peripheral tissues. IL-15-expressing cells have been identified in primary and secondary lymphoid organs using several IL-15 reporter mouse lines, such as IL-15-cyan fluorescent protein (CFP) knock-in mice and IL-15 bacterial artificial chromosome (BAC)-emerald green fluorescent protein (EmGFP) mice (34, 89–91). Various stromal cells with a unique distribution highly express IL-15 *in vivo*, in addition to hematopoietic cells such as macrophages and dendritic cells. IL-15-expressing cells are mainly situated in the thymic medulla, the primary source of which is MHC class II high mTECs. In the bone marrow, IL-15-expressing cells can be found dispersed throughout the marrow cavity, with a fraction of CXCL12-abundant reticular (CAR) cells expressing high levels of IL-15. Within the T-cell zone of the spleen, IL-15-expressing cells are also distributed. In lymph nodes, IL-15 is expressed in all high endothelial venules (HEVs) and a fraction of fibroblastic reticular cells (FRCs) in the T-cell zone and medulla. Intestinal epithelial cells express IL-15 in the intestine. Additionally, blood vascular endothelial cells (BECs) and lymphatic endothelial cells (LECs) upregulate IL-15 expression during inflammatory conditions.

To determine whether distinct IL-15 niches regulate iNKT cell heterogeneity, Cui et al. established an IL-15-floxed mouse model that enables dissection of the local function of IL-15 in various immune microenvironments through crossing with multiple tissue-specific Cre reporter mice (11). The FoxN1-Cre IL-15 conditional knockout (cKO) mice, deficient in IL-15 expression in mTECs, were utilized to elucidate the thymic IL-15 niche for iNKT cells. The FoxN1-Cre IL-15 cKO mice exhibit a reduction in NKT1 cells, but not NKT2 or NKT17 cells, which aligns with a preceding study confirming the indispensability of TEC-derived IL-15 for type 1 innate-like T cells, including iNKT cells (92). Subsequently, mature stage 3 iNKT cells, which are nearly identical to NKT1 cells in mice, can be classified into three subpopulations based on the presence of the NK cell receptor CD244 (also known as 2B4 or SLAMF4) and the chemokine receptor CXCR6: CD244^+^CXCR6^+^ double-positive C2 iNKT cells, CD244^−^CXCR6^+^ single-positive C1 iNKT cells, and CD244^−^CXCR6^−^ double-negative C0 iNKT cells (11). Notably, C2 iNKT cells have nearly vanished, while C1 and C0 iNKT cells have only slightly decreased or stayed the same in the thymus of FoxN1-Cre IL-15 cKO mice. Additionally, although C1 iNKT cells recuperate, C2 iNKT cells remain significantly reduced in peripheral tissues, including the spleen, lung, and liver of FoxN1-Cre IL-15 cKO mice. Furthermore, IL-15 production by mTECs leads to the development and maturation of C0 iNKT cells into C2 and C1 iNKT cells in the thymus. The findings suggest that the development and maturation of C2 iNKT cells rely significantly on the thymic epithelial IL-15 niche. Moreover, a recent study showed that thymic NKT1 cell heterogeneity necessitates TGF-β and IL-15 (93).

Digital RNA-seq showed differential gene expression between two mature thymic iNKT cell subsets, C1 iNKT cells and C2 iNKT cells (11). Besides the NK cell receptor CD244, C2 iNKT cells express KLF2, a transcription factor for NK cell homeostasis (94). KLF2 expression is high in CCR7^+^ iNKT cell progenitors but diminishes during their differentiation into iNKT cell subsets (37). Functionally, C2 iNKT cells express high levels of cytotoxicity-related genes such as IFN-γ and granzymes (*Gzma* and *Gzmb*) at steady state. They also express high levels of chemokines (*Ccl5*), integrins (*Itga1*, also known as *Cd49a*), galectins (*Lgals1*), and multiple killer cell lectin-like receptors (KLRs). Consequently, C2 iNKT cells exhibit properties similar to NK cells. On the other hand, C1 iNKT cells exhibit more T cell-like properties and express genes related to TCR signal strength or T cell activation, including *Icos*, *Lef1*, *Zap70*, and *Cd5*. The heterogeneity of NKT1 in the thymus has recently been identified through scRNA-seq (47), with the NK cell-related signature SLAMF4 (CD244) distinguishing the terminally differentiated NKT1 cell cluster. The SLAMF4^+^ iNKT cell cluster showed high levels of granzyme A and IFN-γ cytotoxic mediators, but not IL-4. Additionally, these Gzma^+^SLAMF4^+^ iNKT cells are detected in peripheral organs.

The dynamic heterogeneity between C2 and C1 iNKT cells in peripheral tissues has been further elucidated (11). Parabiosis experiments revealed that C2 iNKT cells are a newly identified circulating subset of iNKT cells, while C1 iNKT cells are a conventional tissue-resident subset of iNKT cells. The regulation of C2 iNKT cell migration and retention in peripheral tissues is governed by the integrin α1 (CD49a) and S1P receptors S1PR1 and S1PR4, while C1 iNKT cells require CXCR6 for their tissue residency and express high levels of P2RX7. Interestingly, C2 iNKT cells express KLF2, a transcription factor regulating T cell migration by directly controlling cell surface receptors S1PR1 and CCR7 (94, 95). A recent study showed the circulatory signature of iNKT cells, such as *Klf2*, *Cd62l*, *S1pr1*, and *S1pr4* expression, identified through scRNA-seq. Additionally, the study demonstrated that KLF2 regulates the migration and differentiation of iNKT cells by using KLF2-deficient mice (12).

Functionally, C2 iNKT cells play a role in anti-tumor and antiviral immunity (11). Lung metastasis of melanoma cells is exacerbated in mice without C2 iNKT cells, suggesting that conventional tissue-resident C1 iNKT cells alone may be insufficient for mediating anti-tumor immunity. Moreover, rapid immune responses by circulating C2 iNKT cells may be essential for initiating anti-tumor immunity. C2 iNKT cells protect mice from lung metastasis of melanoma cells and show elevated direct tumor-killing capacity in cultures with elevated expression levels of cytotoxic molecules such as IFN-γ, granzyme B, perforin, and TRAIL. Consequently, these C2 iNKT cells circulate throughout the periphery to carry out cancer immunosurveillance and work as a powerful tumor-suppressing subset of iNKT cells. Peripheral C2 iNKT cells express TLR2, IL-1β, and galectin-1 at high levels, which play a crucial role in the immune response against bacteria and influenza viruses (96, 97). Furthermore, they secrete significant amounts of chemokines such as CCL2, CCL5, and CXCL2, which are associated with the recruitment of immune cells and are increased in influenza and coronavirus infections (98). In addition, due to their ability to reduce influenza type A virus (IAV)-induced MDSCs and the restricted maintenance of MDSCs by apolipoprotein E (ApoE), it is presumed that C2 iNKT cells function as a potent MDSC-suppressing subset during IAV infection, facilitated by their high expression of ApoE (81, 99). Consequently, C2 iNKT cells accelerate virus clearance during influenza A virus infection. However, FoxN1-Cre IL-15 cKO mice showed less body weight loss and lower mortality following a sublethal dose of IAV infection. This suggests that circulating C2 iNKT cells play a significant role in regulating the development of IAV-induced acute lung injury, leading to disease progression and increased mortality.

**Figure 1:**
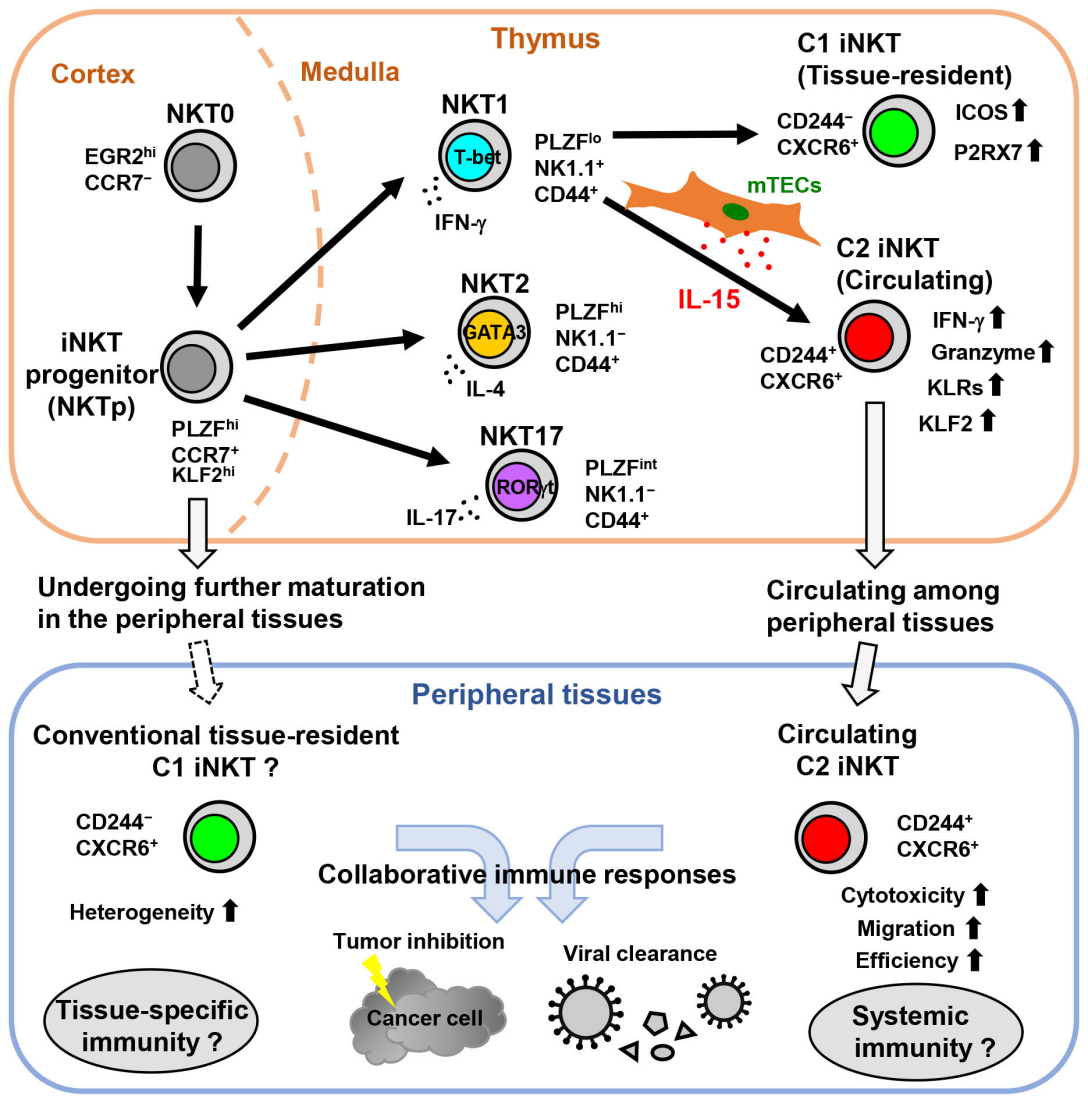
The development of circulating C2 iNKT cells is driven by the thymic epithelial IL-15 niche in mice. In the thymus, NKT0 cells differentiate into CCR7^+^ iNKT cell progenitors (NKTp). NKTp cells can then emigrate from the thymus to peripheral tissues and undergo further maturation or continue their differentiation into effector subsets, including NKT1, NKT2, or NKT17. IL-15 produced by mTECs drives the development and terminal maturation of C2 iNKT cells in the NKT1 cell population. Although most mature iNKT cells in the thymus are tissue-resident, the C2 iNKT cells exhibit high cytotoxicity with NK cell-like features and circulate among peripheral tissues. Subsequently, C2 iNKT cells serve as the circulating iNKT cells in peripheral tissues, whereas C1 iNKT cells are conventional tissue-resident iNKT cells.

Human CD244^+^ C2 iNKT cells have also been found in peripheral blood as counterparts to mouse C2 iNKT cells (11). Human C2 iNKT cells are part of the CD4^−^ iNKT cell fraction that highly expresses genes related to cytotoxicity, such as granzymes, perforin, and granulysin, which are essential for iNKT cell-mediated effector functions in anti-tumor and antiviral immunity. In contrast, human C1 iNKT cells can be found in CD4^+^ and CD4^−^ iNKT cell fractions. In addition, human C2 iNKT cells express CCR5, a chemokine receptor involved in human T cell trafficking (100). Interestingly, in the peripheral blood of aging humans, the frequency of C2 iNKT cells decreases, likely due to their reliance on the thymic microenvironment. Overall, IL-15 expression by mTECs in the thymic microenvironment tightly regulates the development of circulating C2 iNKT cells, while tissue-resident C1 iNKT cells are less reliant on this thymic epithelial IL-15 niche. mTEC-derived IL-15 is important for conferring high cytotoxicity and NK cell-like properties to C2 iNKT cells and contributes to the generation of iNKT cell heterogeneity (**Figure 1**).

Numerous studies utilize agonists such as α-galactosylceramide or PMA/ionomycin to stimulate iNKT cells and evaluate their functional potential. However, the regulation of activation and functional heterogeneity of iNKT cells *in vivo* remains unresolved. As the circulating and tissue-resident iNKT cells seem to have different dependencies on the IL-15 niche in individual tissue, combining the IL-15-floxed mouse model with multiple tissue-specific Cre reporter mice may be a useful tool for dissecting the functional heterogeneity of tissue-resident iNKT cells, especially NKT1 cells *in vivo*.

## Heterogeneity of iNKT cells in cancer immunotherapy

Due to their diverse immunoregulatory functions in innate and adaptive immunity against cancer and infection, iNKT cells are considered an attractive candidate for cancer immunotherapy. In addition, iNKT cells offer a novel platform and approach to enhance chimeric antigen receptor (CAR)- or engineered TCR-based cancer immunotherapy. An iNKT cell-targeted immunotherapy has been reported to successfully treat advanced stages of non-small cell lung cancer and head and neck cancer (101). Clinical trials using activated iNKT cell adoptive transfer in non-small cell lung cancer have reported no serious adverse effects and have shown an increase in circulating iNKT cell numbers. However, few patients experienced a reduction in tumor progression, likely due to the functional defects and absent long-term efficacy of iNKT cells in cancer patients. Subsequently, patients who receive combined therapy with α-GalCer-loaded DCs, which can enhance iNKT cell activation, show increases in circulating iNKT cell numbers and IFN-γ production, with some patients achieving a partial anti-tumor response (102–104). Prior studies have also demonstrated the effective redirection of human iNKT cells towards hematologic or solid malignancies by engineering these cells to express tumor-specific CARs or TCRs (105, 106). TCR-engineered iNKT (TCR-iNKT) cells that use a second TCR for a tumor-associated peptide create bispecific effectors for CD1d- and MHC-restricted antigens and show higher efficacy in inhibiting the progression of multiple tumors expressing the cognate antigen compared to CD8^+^ T cells engineered with the same TCR (107).

To date, iNKT cells have been isolated from peripheral blood mononuclear cells (PBMCs) of patients and expanded ex vivo before adoptive transfer into the patient. However, iNKT cell-based immunotherapy faces several significant challenges, such as the limited number of iNKT cells obtained from peripheral blood, weak activity, and low tissue specificity of ex vivo expanded iNKT cells. To enhance the effectiveness and tissue-specificity of iNKT cell-based immunotherapy across various tissues, it is necessary to examine the diversity of tissue-resident and circulating iNKT cells. For instance, the human CD244^+^ C2 iNKT cells, which correspond to the circulating C2 iNKT cells in mice, exhibit robust cytotoxicity owing to their high expression of granzyme B, perforin, and granulysin. Utilizing this subset of iNKT cells may amplify the therapeutic efficacy of iNKT cell-based immunotherapy. Thus, depending on the type of target cancer, chemokine receptors for migration, cytokines, and co-stimulatory factors for survival, proliferation, and activation of tissue-resident or circulating iNKT cells can be utilized.

Graft-versus-host disease (GVHD) is a common and serious complication of allogeneic transplantation and CAR-T cell immunotherapies. Recipient tissue cells express high levels of MHC class I and II molecules, resulting in broad binding of donor T cells with highly variable TCRs. iNKT cells, on the other hand, can escape MHC recognition because they are not restricted by MHC, unlike T cells. iNKT cells have been shown to modulate and reduce the GVHD response in transplant patients. For instance, it has been proposed that CD4^+^ iNKT cells control GVHD via their immunoregulatory function through IL-4 (108). A recent study used scRNA-seq to analyze iNKT cells from healthy individuals and GVHD patients (109). The findings revealed that CD4^−^CD94^+^ iNKT cells with substantial cytotoxic potency are associated with GVHD control, possibly by inducing DC death. Notably, all human C2 iNKT cells are CD4^−^, and substantial cytotoxicity is observed due to elevated CD94 (also known as KLRD1) levels, similar to the CD4^−^CD94^+^ iNKT cells. These cells show potential as a platform for CAR-NKT or engineered TCR-NKT cells.

Immunotoxicity is an adverse effect of drugs like immune checkpoint inhibitors on the immune system. It frequently arises in organs with abundant tissue-resident lymphocytes, such as the liver, lung, and intestine (110, 111). The mechanism underlying tissue-specific immunotoxicity is largely unknown, making it challenging to predict. Because tissue-resident iNKT cells are enriched in these tissues and change with age, understanding the heterogeneity of iNKT cells may offer a new approach to evaluate and reduce tissue-specific drug-induced immunotoxicity.

A phase I clinical trial investigated autologous iNKT cells that co-express a GD2-specific CAR with IL-15 (GD2-CAR-NKT) against neuroblastoma. The trial showed that CAR-NKT cells express elevated levels of CD62L, and responders have a higher abundance of CAR-NKT cells with elevated KLF2 expression than non-responders (112). Furthermore, GD2-CAR-NKT cells exhibit similar or reduced toxicity, such as cytokine release syndrome (CRS), compared to conventional GD2-CAR T cells targeting the same antigen in neuroblastoma patients. These results suggest that a specific subset of iNKT cells may primarily impact the efficacy of CAR NKT cell immunotherapy. Besides the highly cytotoxic nature of human C2 iNKT cells, they express high levels of KLF2. Therefore, understanding the heterogeneity of iNKT cells could assist the direction of CAR-NKT cell engineering for various cancer types and offer valuable biomarkers for predicting the efficacy and immunotoxicity of iNKT cell-based immunotherapies.

## Conclusion

iNKT cells exhibit heterogeneity during their developmental stages and depending on their location within various tissues. This heterogeneity arises from the differential expression of transcription factors, as well as distinctive gene expression profiles linked to iNKT cell kinetics, distribution, and effector functions within different tissues. IL-15 production by mTECs in the thymic microenvironment contributes to the acquisition of heterogeneity among iNKT cells. In mice, the thymic epithelial IL-15 niche strictly controls the development of a novel circulating subset of NKT1 cells called C2 iNKT cells (**Figure 1**). These C2 iNKT cells express high levels of cytotoxic molecules and exhibit more NK cell-like characteristics, while the conventional tissue-resident C1 iNKT cells exhibit more T cell-like characteristics. In addition, human C2 iNKT cells with high cytotoxicity have also been identified in peripheral blood as counterparts to mouse C2 iNKT cells. Understanding the heterogeneity of iNKT cells could offer a new strategy to enhance and predict the efficacy of iNKT cell-based immunotherapies and to assess and reduce tissue-specific immunotoxicity.

## Author contributions

GC: Conceptualization, Funding acquisition, Writing – original draft, Writing – review & editing. SA: Writing – review & editing. RK: Writing – review & editing. KI: Conceptualization, Funding acquisition, Writing – review & editing, Writing – original draft.
